# An Extremely Rare Cause of Bruising in Children: Autoerythrocyte Sensitization Syndrome

**DOI:** 10.5505/tjh.2012.67878

**Published:** 2012-06-15

**Authors:** Mesut Okur, Hakan Turan, Aybars Özkan, Cemalettin Güneş, Kenan Kocabay

**Affiliations:** 1 Düzce University, School of Medicine, Department of Pediatrics, Düzce, Turkey; 2 Düzce University, School of Medicine, Department of Dermatology, Düzce, Turkey; 3 Düzce University, School of Medicine, Department of Pediatric Surgery, Düzce, Turkey

## TO THE EDITOR

Autoerythrocyte sensitization syndrome (ASS)—also known as Gardner-Diamond syndrome—is an autoimmune vasculopathy associated with sensitization to phosphatidylserine, a phosphoglyceride of red blood cell membranes [1]. The syndrome was first described in 1955 by Gardner and Diamond in 4 female patients [2]. ASS is typically seen in adult females; however, pediatric and male patients have also been described [2,3,4]. The disease usually develops after psychic stress. It is characterized by development of painful edematous skin lesions that progress to ecchymoses during the following 24 h [5]. The diagnosis of ASS is confirmed via an autoerythrocyte sensitization test [2]. Herein we report ASS in a previously healthy 7-year-old boy. 

The patient presented to the emergency department with spontaneous onset of acute pruritic, painful, edematous ecchymosis on both lower extremities. According to anamnesis, the lesions began as painful edematous plaques, and eventually became purplish. History of trauma and infection was negative. The patient was not taking any medications and his medical history was unremarkable. The patient’s mother had a history of recurrent spontaneous bruising lesions similar to those observed in the patient. Physical examination showed that his vital signs were within normal limits. His weight and height were in the 50^th^ and 75^th^ percentile, respectively, for his age group. 

**Figure 1:**
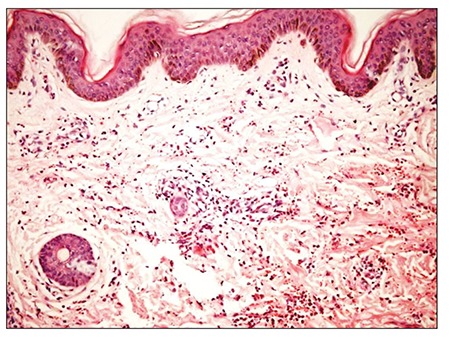
Normal epidermis and mild edema, extravascular erythrocytes, and nonspesific inflammatory cell infiltration in dermis.

**Figure 2:**
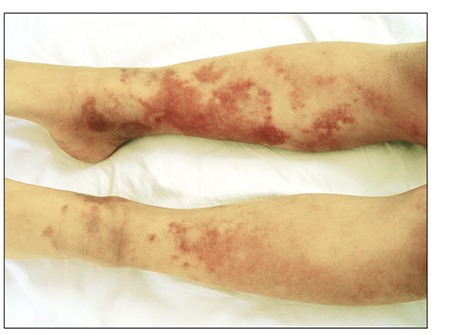
Red and purple-colored, slightly infiltrated, geographic patterned ecchymotic patches in various sizes on below-kneeregion.

Red and purple, slightly firm, irregularly shaped ecchymotic patches varying in size were observed below the knees of both legs (Figure 1). Other systemic examinations were normal. The complete blood count and differential were normal. The erythrocyte sedimentation rate was 8 mm h^–1^ and C-reactive protein was negative. Coagulation studies showed that the prothrombin time, partial thromboplastin time, bleeding time, factor VIII, fibrinogen, D-dimer, anti-thrombin III, protein C, and S levels were normal. Antinuclear antibodies, anti-double-stranded DNA, anticardiolipin antibodies, lupus anti-coagulant, and Coombs’ tests were negative. Hepatitis A, B, and C, toxoplasma, rubella, and cytomegalovirus serology findings were negative. Histopathological examination showed normal epidermis and mild edema, extravascular erythrocytes, and non-specific inflammatory cell infiltration in the dermis. There was no evidence of vasculitis (Figure 2). Written informed consent was obtained from the patients’ parents. 

We intradermally injected 0.1 mL of the patient’s own red blood cells into his right forearm and there was only a mild reaction to the test. No medications were given to the patient, except for a single dose of dexamethasone and pheniramine, which were administered upon admission tohospital. The patient was given bed rest. On d 5 of hospitalization the rashes became pale and over the course of the next 5 d disappeared completely. The patient was then discharged and knowledge was given that this benign disorder would recur especially due to stresses to his parents. No dermal findings or patient complaints were noted at the 10-d post-discharge follow-up. 

ASS is a chronic, unexplained syndrome that presents with puzzling signs and symptoms [6]. ASS is characterized by painful ecchymotic lesions and is most commonly observed in women experiencing emotional stress or psychiatric disorders [2]. Although ASS is seen usually in adult women, pediatric cases have been reported [3,6]. In the majority of cases the lesions appear when patients experience severe emotional stress [7,8]. This disorder was named as psychogenic purpura based on its association with psychiatric disturbance in the majority of patients [9]. It should be noted, though, that some patients do not have any specific psychopathological syndrome. The presented patient was introverted and shy. Bruises on his legs appeared 1 d after having an argument with his mother and father. 

ASS lesions are characterized by sudden onset, pain, swelling, bleeding, and variable size, and can occur on the skin of any part of the body [8]. The lesions vary in size from 1-2 cm in diameter to involvement of an entire limb. The lesions typically occur on the extremities and rarely on less accessible locations, such as the back. The lesions are preceded by parasthesia or pain [5]. The lesions are recurrent, usually resolving within a period of 2 weeks [7]. 

The most common histological features of ASS are erythrocyte extravasations, dermal edema, and perivascular inflammation [10]. As in the presented patient, there are no specific laboratory anomalies in ASS patients. Hematological parameters, including hemoglobin, hematocrit, platelet count, peripheral smear, erythrocyte sedimentation rate, electrolytes, bleeding time, prothrombin, thrombin, and partial thromboplastin time, and coagulation factors are usually within normal limits. Laboratory signs of systemic disorders are absent. A reliable diagnostic test for ASS consists of intracutaneous injection of 1 mL of 80% suspension of washed erythrocytes obtained from the patient [2]. Ratnoff reported that the skin test was positive in only 59% of patients tested in his series [7]. The skin test may also be negative in pediatric cases [3]. The presented patient had only a mild reaction to the ASS skin test. 

A large number of pharmacologic agents and intervention have been used to treat ASS, including antihistamines, albumin infusions, corticosteroids, chemotherapy, antidepressants, hormones, vitamin C, and splenectomy [11]; however, none has proved to be of significant benefit in controlling the manifestations of the disease [11]. No medical treatment was administered to the presented patient, other than a single dose of steroid and antihistaminic upon admittance to the hospital. The patient’s bruises, pain, and swelling disappeared completely during 10 d of bed rest. 

The prognosis of ASS is good and no deaths have been reported due to this syndrome or its complications [5,7]. In some individuals the syndrome may remit for months or years and recur at a time of severe emotional stress [7]. In the majority of patients, relapses may occur in the future after the lesions resolved. However, remissions even may be stable for many years. In fact, in the presence of specific histological changes, positive intracutaneous test results, psychic disorder, onset of lesions associated with stress, and the absence of hematological disorders or systemic diseases, it is not difficult to diagnose ASS. 

In conclusion, ASS is a rare syndrome most typically observed in adult females with psychological disturbances, but it is important to be aware that ASS can also occur in children. Despite the severe presentation of the disease, prognosis is fairly good. ASS should be considered in the differential diagnosis of purpura and ecchymosis in children. 

**Conflict of Interest Statement **

The authors of this paper have no conflicts of interest, including specific financial interests, relationships, and/or affiliations relevant to the subject matter or materials included.
